# rGAI: An R package for fitting the generalized abundance index to seasonal count data

**DOI:** 10.1002/ece3.9200

**Published:** 2022-08-22

**Authors:** Emily B. Dennis, Calliste Fagard‐Jenkin, Byron J. T. Morgan

**Affiliations:** ^1^ Butterfly Conservation Dorset UK; ^2^ School of Mathematics, Statistics and Actuarial Science University of Kent Kent UK; ^3^ Centre for Research into Ecological and Environmental Modelling University of St Andrews St Andrews UK

**Keywords:** flight period, generalized abundance index, multivoltine, phenology, seasonal abundance, stopover model

## Abstract

The generalized abundance index (GAI) provides a useful tool for estimating relative population sizes and trends of seasonal invertebrates from species' count data and offers potential for inferring which external factors may influence phenology and demography through parametric descriptions of seasonal variation. We provide an R package that extends previous software with the ability to include covariates when fitting parametric GAI models, where seasonal variation is described by either a mixture of Normal distributions or a stopover model which provides estimates of life span. The package also generalizes the models to allow any number of broods/generations in the target population within a defined season. The option to perform bootstrapping, either parametrically or nonparametrically, is also provided. The new package allows models to be far more flexible when describing seasonal variation, which may be dependent on site‐specific environmental factors or consist of many broods/generations which may overlap, as demonstrated by two case studies. Our open‐source software, available at https://github.com/calliste‐fagard‐jenkin/rGAI, makes these extensions widely and freely available, allowing the complexity of GAI models used by ecologists and applied statisticians to increase accordingly.

## Graphical abstract

We describe an R package for the analysis, primarily, of data on seasonal insects. It is flexible and user‐friendly and provides estimates of important characteristics relating, for example to the timing of events: phenology. This allows the determination of temporal changes, and the effects of climate change.

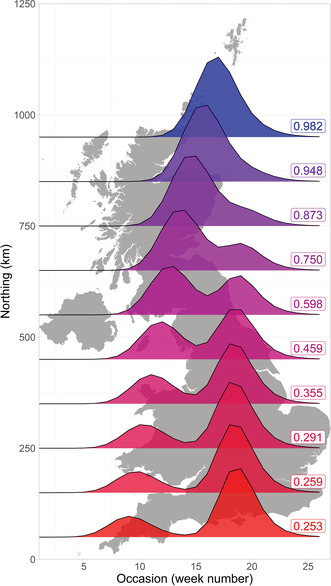

## 1 INTRODUCTION

Suitable statistical models are vital for monitoring species' populations at a time of climate change, habitat degradation, and consequent major loss of biodiversity. The generalized abundance index (GAI, Dennis et al., 2016) provides a useful tool for modeling count data which exhibit variation in numbers within a season, and can take several different forms, including parametric and non‐parametric alternatives. The development of the GAI was motivated by an application to invertebrates, specifically butterflies, which have multistage life cycles, where counts are usually only made of the most visible adult stage. Counts within a season typically fluctuate as individuals emerge, reproduce and then die off, with one or more generations/broods of adults apparent per year. Long‐term monitoring schemes often consist of many sites over many years, and previous modeling approaches were often time‐consuming to run. The need for more efficient data analysis motivated the development of the GAI, which also offers flexibility in describing seasonal variation in count data.

Here, we outline the GAI approach; full details are provided in Dennis et al. (2016). Within a single year, suppose that counts are recorded at *S* sites each visited on at most *T* occasions. Each count *y*_*i*,*j*_ for the *i*th site and *j*th visit is regarded as a realization of a discrete random variable, for example Poisson (alternative distributions are described later), with expectation

$$\lambda_{i,j}=N_{i}a_{i,j}$$

where *N*_*i*_ represents relative total abundance for site *i*, and *a*_*i*,*j*_ denotes a function describing seasonal variation in counts in terms of a small set of parameters. Estimates of abundance are relative since not all individuals present during a visit will be observed, and detection is assumed to be constant (but see Matechou et al., 2014). Variation in transect length is also not accounted for, but could be by appropriately scaling *N*_*i*_. The GAI encompasses three options for *a*_*i*,*j*_ which describe seasonal variation in counts:

- Splines—seasonal variation is described by a flexible curve representing *a*_*i*,*j*_, for example using B‐splines (Dennis et al., 2016). The *a*_*i*,*j*_ are scaled to sum to unity, such that they describe how *N*_*i*_ is spread over the season. The seasonal curve is typically assumed to be the same across all *S* sites and the smoothness of the curve is defined by the number of knots for the spline. This option corresponds closely with the method previously developed for modeling butterfly count data (Dennis et al., 2013) and is the approach typically used for abundance trend reporting—see for example Brereton et al. (2020); Fox et al. (2021).
- Mixture model—seasonal variation is taken as a mixture of *B* Normal probability density functions (corresponding to one or more broods within a year) so that

$$a_{i,j}=\sum_{b=1}^{B}w_{i,b}\frac{1}{\sigma_{i,b}\sqrt{2\pi }}\exp\left\{-\frac{{\left(t_{i,j}-\mu_{i,b}\right)}^{2}}{2\sigma_{i,b}^{2}}\right\},$$

where *t*_*i*,*j*_ denotes the *j*th occasion, which is the time during the season typically measured by day or week, and *w*_*i*,*b*_, *μ*_*i*,*b*_ and *σ*_*i*,*b*_ correspond to the weight, mean, and standard deviation, respectively, for the *i*th site and *b*th brood, and $\sum_{b=1}^{B}w_{i,b}=1$, *B* ≥ 1. The weights *w*_*i*,*b*_ describe the relative sizes of the *B* broods with respect to each other. As in the spline case, the mixture model is a phenomenological model, where the *a*_*i*,*j*_ effectively describe how *N*_*i*_ is spread over time, where *a*_*i*,*j*_ integrate to unity.

- Stopover model—this is based upon the model proposed in Matechou et al. (2014) which incorporates parameters relating to butterfly life span, that is, lifetimes of individual adult butterflies, by estimating survival probabilities. In brief,

$$a_{i,j}=\sum_{d=1}^{j}\beta_{i,d-1}\left(\prod_{k=d}^{j-1}\phi_{k,c}\right)$$

for *j* = 1,…,*T* and *c* = *k* − *d* + 1, where *β*_*i*,*d*‐1_ are the proportions of individuals emerging at visit *d*, such that $\sum_{d=1}^{T}\beta_{i,d-1}=1$ for site *i*, which are described by appropriate areas under a mixture of *B* Normal densities,

$$\beta_{i,d-1}=\sum_{b=1}^{B}w_{i,b}\left\{F_{i,b}\left(t_{i,d}\right)-F_{i,b}\left(t_{i,d}-1\right)\right\},$$

where $F_{i,b}\left(t_{i,d}\right)=\mathrm{Pr}\left(X\leq t_{i,d}\right)$ for $X\sim N\left(\mu_{i,b}\sigma_{i,b}^{2}\right)$, with mean emergence date *μ*_*i*,*b*_, standard deviation *σ*_*i*,*b*_, and weighting *w*_*i*,*b*_. *ϕ*_*k*,*c*_ is the probability an individual present for *c* occasions and present at visit *k*, will remain until visit *k* + 1. Since *ϕ*_*k*,*c*_ represents apparent survival probability from one visit to the next, adult life spans may be estimated by 1∕(1−*ϕ*_*k*,*c*_). Unlike the spline and mixture models, the stopover model proposes a mechanism, of which the *N*_*i*_ are a part of. Hence, the model results in complex bounds on the *a*_*i*,*j*_, where it is the emergence parameters *β*_*i*,*d*‐1_ which sum to unity. See Matechou et al. (2014) for full details of this model.

Where counts, *y*_*i*,*j*_, are assumed to be Poisson, efficient model fitting of the GAI is achieved by maximizing a concentrated (or profile) likelihood with respect to only the parameters associated with {*a*_*i*,*j*_} and estimating each *N*_*i*_ by suitably scaled site totals. An iterative approach is taken when assuming negative binomial and zero‐inflated Poisson distributions, as explained in Dennis et al. (2016).

To date, the GAI has primarily been adopted as a method for producing species' abundance trends—for example, it is used annually for reporting of UK butterfly trends (Brereton et al., 2020), which contribute to UK biodiversity indicators (Department for Environment, Food and Rural Affairs, UK, 2020). The approach has also been used in status assessments for larger moths in Britain (Fox et al., 2021; Randle et al., 2019), and in analyses of European butterfly populations (Van Swaay et al., 2020). Where the specific goal is to produce abundance trends, the spline option for describing seasonal variation is used. Here, flight periods are typically assumed to be fixed over sites (as originally developed using generalized additive models, Dennis et al., 2013), or geographical subsets of them (Schmucki et al., 2016).

However, the GAI presents wider opportunities for further insights into seasonal count data, particularly through the application of the parametric descriptions of seasonal variation from the mixture and stopover models. The rGAI package therefore extends previous software to provide accessible code that can accommodate the inclusion of relevant covariates, as well as any number of broods within a defined season. The package also provides the opportunity for wider exploration of stopover models, including estimating species' life spans.

## 2 rGAI PACKAGE OVERVIEW

The rGAI package extends the software made available in the supplementary materials of Dennis et al. (2016). Model fitting is by maximum‐likelihood, and model parameters are transformed using combinations of logarithmic (e.g., for *μ* and *σ*) or logistic (e.g., for *w* and *ϕ*) link functions. rGAI functions with simple inputs allow survey data to first be verified (for duplicate or missing entries across time, or across sites). Then, initial values can be selected for the model fitting process, accounting for the appropriate link scale. The GAI with the mixture or stopover model description for seasonal variation can be fitted with covariate inclusion for parameters of interest, and measures of uncertainty on parameter estimates can be produced using bootstrapping methods. Table 1 details the most important functions in the rGAI package and provides a brief description of their intended use. The rGAI package also includes a markdown vignette file, with a tutorial‐style presentation of all of the package's functionality, as well as installation instructions. The latest version of rGAI is made freely available at https://github.com/calliste‐fagard‐jenkin/rGAI.

**TABLE 1:** Description of key functions in the rGAI package.

Function	Description
extract_counts	Extracts a table of counts across sites and occasions from an input data.frame, to facilitate data cleaning and visualization
transform_starting_values	Produces a set of initial parameter values on the link scale, given user inputs on the parameter scale
fit_GAI	Fits GAI models, with any number of broods, with a spline, mixture model, or stopover model to describe seasonal variation. Counts can be modeled with a negative binomial, Poisson, or zero‐inflated Poisson distribution
bootstrap	Produces bootstrap confidence intervals for all parameters by either resampling them from their asymptotically Normal distribution (parametric bootstrap), or re‐fitting models by resampling sites (non‐parametric bootstrap). Bootstraps can be provided on the link or parameter scale
transform_output	Transforms parameter estimates from the link scale to the parameter scale, with custom covariate values, or those observed in the data
transform_bootstrap_parameters	Transforms bootstrap confidence intervals of parameter values from the link scale to the parameter scale, for custom covariate values
plot	Produces plots of fitted GAI models, with the option of scaling curves by the site total, or producing plots showing variation between sites due to covariate values
AIC	Extracts Akaike's information criterion for a fitted GAI model
summary	Produces a summary of a fitted GAI model, with parameter estimates and standard deviations for parameters, on the link scale

Covariates can be included in the linear predictors for the mean emergence date of individuals {*μ*_*i*,*b*_}, the standard deviation of each component {*σ*_*i*,*b*_}, and the weightings {*w*_*i*,*b*_}. Covariate formulae can be specified individually for each brood, or be shared by all broods, for all count distributions (Poisson, negative binomial, or zero‐inflated Poisson). The package flexibly allows any number of broods within a season, and includes appropriate custom link functions for the relevant parameters, for example to ensure that for the case *B* = 3, *μ*_1_ < *μ*_2_ < *μ*_3_ and *w*_1_ + *w*_2_ + *w*_3_ = 1.

The package vignette, as well as two examples below, provide concrete examples of how this flexibility may be used to produce models that are more representative of underlying species biology than was possible with previous available software.

## 3 EXAMPLES

We now demonstrate usage of the rGAI package via two examples. These examples are presented to illustrate the general capabilities of the rGAI package for modeling seasonal count data, rather than providing full statistical analyses.

Both examples feature seasonal count data for 2018 from the UK Butterfly Monitoring Scheme (UKBMS, Brereton et al., 2020). The scheme consists of a long‐running network of transects at which counts of butterflies are made on a weekly basis from April to September under standardized, favorable weather conditions (Pollard & Yates, 1993).

### 3.1 Incorporating covariates

We illustrate the usage of covariates in the rGAI package, to allow for seasonal variation in counts to vary over space, by application to data for the Common Blue, *Polyommatus icarus*. This species is known to be bivoltine in the south of the UK, with two generations of adult butterflies per season, but univoltine further north, with a single peak in counts per season, as discussed in Asher et al. (2001, p. 47), and demonstrated by Matechou et al. (2014) using a stopover model.

**FIGURE 1:**
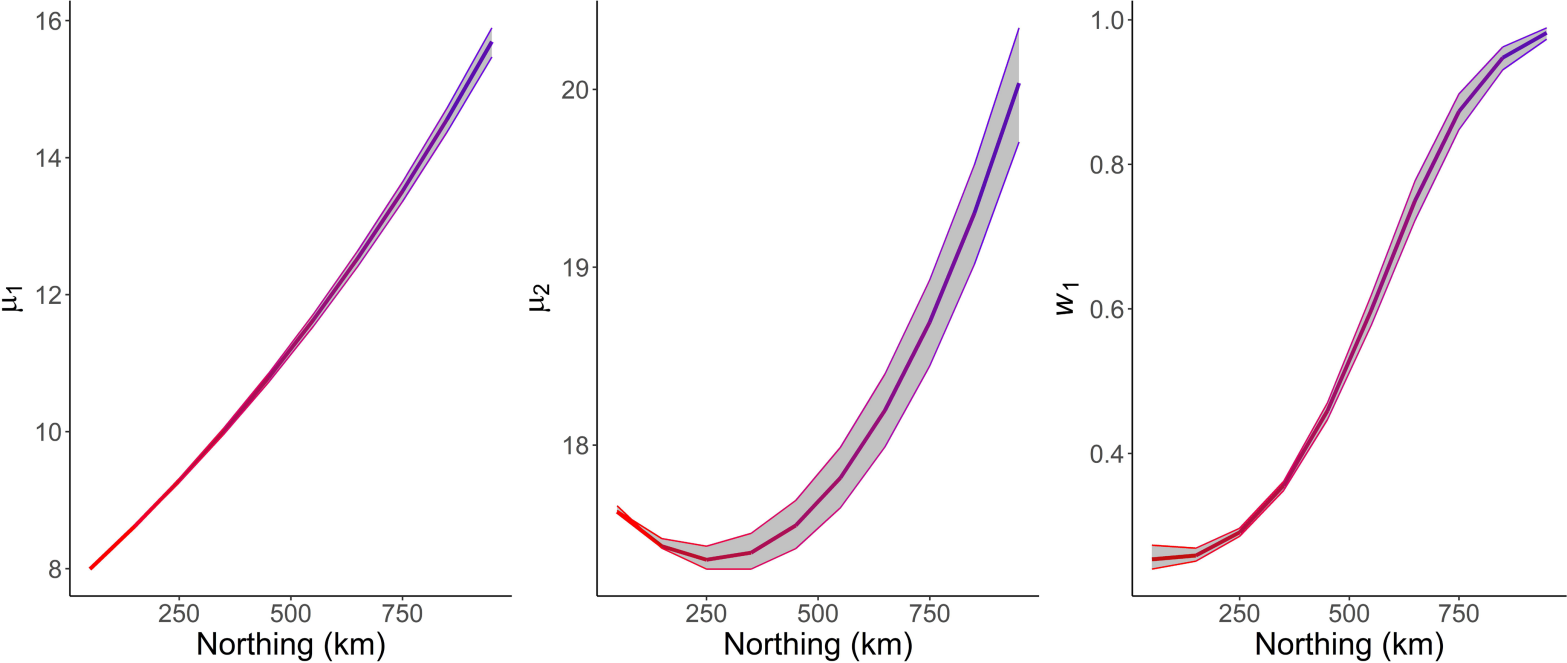
Parameter estimates of mean emergence times, *μ*_1_ and *μ*_2_, and mixing probability, *w*_1_, from fitting the GAI with Poisson distribution and a stopover model to counts of the Common Blue butterfly in 2018, with varying northing. For *μ*_1_ and *μ*_2_, week 1 corresponds to the start of April. 95% confidence intervals derived by parametric bootstrap are shown.

**FIGURE 2:**
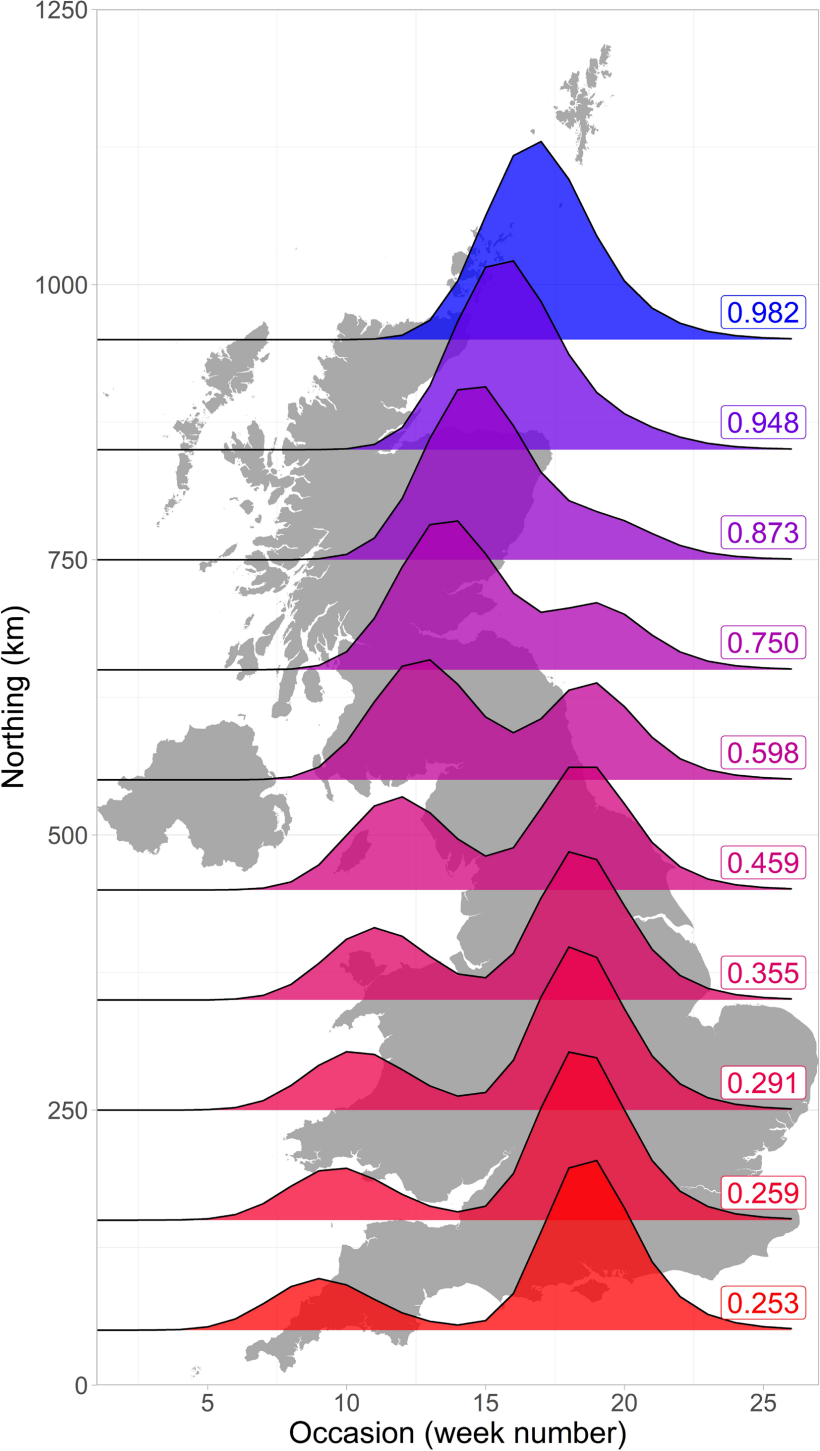
Estimated seasonal pattern for a sample of northing values (each 100 km, from 50 to 950 km) from fitting the GAI with Poisson distribution and a stopover model to counts of the Common Blue butterfly in 2018. The area under the curve is the same for each northing value. The estimate of the mixing probability, *w*_1_, which describes the size of the first brood relative to the second, is given for each northing value at 100 km intervals. Week 1 corresponds to the start of April.

Here, the rGAI package was used to fit a GAI with Poisson distribution and a stopover model to describe seasonal variation, where mean emergence dates were regressed linearly on northing for both broods, and the weighting parameter was described as a quadratic function of northing. Parameter estimates are given in Table 2, with constant survival probability, *ϕ*, and constant standard deviations for each brood (*σ*_1_ and *σ*_2_). The transform_output function was used to produce parameter estimates on the parameter scale for specified covariate values (northing), which are shown in Figure 1, as well as estimates of seasonal pattern, which are presented in Figure 2. Figures 1 and 2 illustrate the gradual delay in mean emergence date with increasing northing, along with the increasing closeness of the two broods, until the species' seasonal pattern shows only one generation in the most northern parts of the UK.

**TABLE 2:** Parameter estimates (with standard errors, SE) for the GAI, fitted with Poisson distribution and stopover model, applied to UK count data for the Common Blue butterfly in 2018, where *μ*_1_ is the mean emergence for the first brood, *μ*_*d*_ is the difference between mean emergence times *μ*_1_ and *μ*_2_, and *w*_1_ is the weighting of the size of the first brood with respect to the second brood, such that *w*_1_ + *w*_2_ = 1.

Parameter	Estimate	SE
$\mu_{1}\left(\text{intercept}\right)$	2.334	0.003
$\mu_{1}\left(\text{northing}\right)$	0.142	0.002
$\mu_{d}\left(\text{intercept}\right)$	1.964	0.004
$\mu_{d}\left(\text{northing}\right)$	−0.168	0.003
$w_{1}\left(\text{intercept}\right)$	−0.438	0.019
$w_{1}\left(\text{northing}\right)$	0.790	0.020
$w_{1}\left({\text{northing}}^{2}\right)$	0.241	0.018
*σ*_1_	1.572	0.017
*σ*_2_	1.292	0.026
*ϕ*	0.468	0.009

*Note*: As they vary with northing, estimates for *μ*_1_, *μ*_*d*_ and *w*_1_ are shown on the link scale (log link for *μ* and logistic link for *w*_1_). See estimates on the parameter scale in Figure 1. Estimates for the standard deviation of the emergence period for each brood, *σ*_1_ and *σ*_2_, and weekly survival probability, *ϕ*, are constant, and therefore shown on the parameter scale.

This brief example is based upon data for a single year, but application to multiple years offers the potential to assess analytically how the change from two to one broods might vary over time, for example due to climatic factors, as well as consider whether changes in abundance for different broods are in synchrony (Asher et al., 2001, p. 162).

By fitting the GAI with a stopover model, an estimate of survival was also obtained ($\hat{\phi }=0.47)$, with a 95% confidence interval of (0.45, 0.49), produced by a parametric bootstrap. Application to multiple years would allow for assessment of potential changes in species' lifespans over time, as demonstrated for two butterfly species in Dennis et al. (2016).

### 3.2 Modeling multiple broods

The mixture and stopover model formulations of the GAI are described in Dennis et al. (2016) in terms of any general number of broods/generations, *B*, within a year/season, but to date, code for a general number of broods has not been widely available; hence, applications have been limited to a maximum of *B* = 2, with the exception of a small simulation example for the stopover model in Matechou et al. (2014) which considered values up to *B* = 3. The rGAI package extends existing code to allow for any number of broods.

**FIGURE 3:**
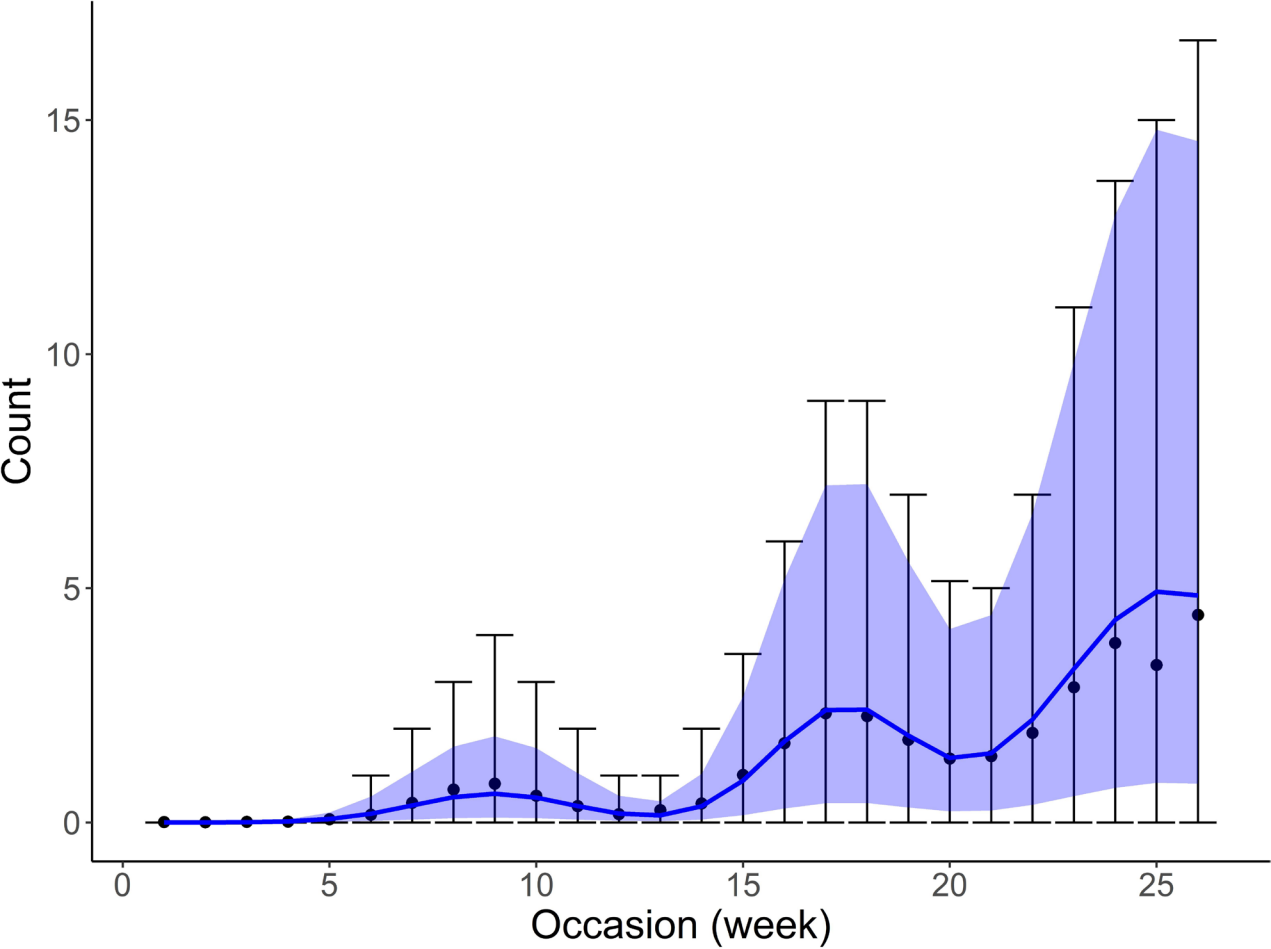
Observed mean count per week (black circles), averaged over sites, with 5% and 95% quantiles of all observed counts shown as error bars, for the Small Copper butterfly in 2018. The predicted mean count per week, averaged over sites, is shown in blue, along with predicted 5% and 95% quantiles for comparison. Predicted values are estimates from the best‐fitting model from Table 3, for which parameter estimates are given in Table 4. Week 1 corresponds to the start of April.

Here, we demonstrate application of the package to data for Small Copper, *Lycaena phlaeas*, for which the overall seasonal pattern suggests three broods within a year, with the third brood at the end of the monitoring season (Figure 3). Using the mixture model formulation, we fit and compare several models for varying *B* = 1, 2, 3, as well as different distributions (Poisson, zero‐inflated Poisson and negative binomial), and fixed or individual standard deviations, *σ,* which describe the variation in flight period lengths. Note that this was not an exhaustive model comparison.

Based on AIC, the model correctly identifies the species as having three broods (Table 3) and indicates the negative binomial distribution to be the most suitable, as well as individual estimates of *σ*, for each brood, suggesting that the time period for each brood varies in length. Although we use AIC for illustration here, alternative model selection approaches could be used, such as other information criteria or tests based directly on likelihood values.

**TABLE 3:** Model comparison for selected GAI fitted with mixture models applied to counts for the Small Copper butterfly, where *n* is the number of model parameters.

Model	*n*	AIC	ΔAIC
P, *B* = 1	2	42,075	11,431
P, *B* = 2, *σ*_shared_	4	42,081	11,437
P, *B* = 3, *σ*_shared_	6	39,488	8844
ZIP, *B* = 3, *σ*_shared_	7	35,731	5087
NB, *B* = 3, *σ*_shared_	7	30,665	22
NB, *B* = 3, *σ*_1,2,3_	9	30,644	0

*Note*: Models are defined by the distribution used (P = Poisson, ZIP = zero‐inflated Poisson, NB = negative binomial), the number of broods *B*, and, for *B* > 1, whether *σ*, the standard deviation for the flight period curves, are shared across broods or estimated per brood. AIC denotes the Akaike information criterion and ΔAIC denotes the difference for each model between its AIC value and the smallest AIC value in the set of fitted models. The best model corresponds to ΔAIC = 0.

Transformed parameter estimates for the best‐fitting model are given in Table 4, along with 95% confidence intervals produced using a parametric bootstrap. Using a bootstrap also allows for the production of a confidence interval for other quantities of interest, for example, the estimated seasonal pattern, *a*_*i*,*j*_, estimates of relative site abundance, *N*_*i*_, and predicted counts, avoiding the use of the delta method which is likely to be complex in these cases.

**TABLE 4:** Transformed parameter estimates for the best‐fitting GAI model (based on the AIC values given in Table 3) applied to counts for the Small Copper butterfly.

Parameter	Estimate	Lower	Upper
*μ*_1_	8.970	8.839	9.102
*μ*_2_	17.437	17.307	17.564
*μ*_3_	25.388	25.222	25.550
*σ*_1_	1.911	1.814	2.016
*σ*_2_	1.711	1.615	1.810
*σ*_3_	2.596	2.414	2.808
*w*_1_	0.064	0.060	0.068
*w*_2_	0.229	0.214	0.244
*r*	0.830	0.786	0.880

*Note*: 95% confidence intervals are provided based on a parametric bootstrap. The means and standard deviations of the flight period are denoted by *μ*_*b*_ and *σ*_*b*_, for each brood *b*. *w*_1_ and *w*_2_ describe the weighting of the size of the first and second brood, where *w*_1_ + *w*_2_ + *w*_3_ = 1. *r* is the dispersion parameter for the negative binomial distribution.

This example demonstrates the potential of the GAI for modeling species with more than two generations within a season, and wider application could again involve extension to analysis over multiple years, as well as the incorporation of relevant covariates to account for spatial variation as in the previous example.

## 4 DISCUSSION/FUTURE AVENUES

The rGAI package has been designed to provide easy‐to‐use software for fitting the GAI, particularly with parametric descriptions of seasonal variation through mixture and stopover models. The ability to include covariates flexibly into parameters of interest offers the potential for further studies and improved understanding of spatio‐temporal variation in species' phenology. Hodgson et al. (2011) considered variation in phenology over space and time using generalized additive models, but through parametric descriptions of seasonal variation, the GAI can provide simple phenological summaries from parameters of interest, as well as separately for each brood, thus offering opportunities beyond many previous phenology studies which have been limited to species exhibiting a single peak in abundance or to the first generation only (Bell et al., 2019; Macgregor et al., 2019; Roy et al., 2015).

Generalization to accommodate any number of broods/generations within a season provides the opportunity for application of the GAI to species which are known to exhibit more than two broods per year, as well as to species with a less predefined number of broods, which may vary over space and time when species show phenotypic plasticity in voltinism and phenology (Macgregor et al., 2019). The rGAI package provides opportunities to better test for and identify such variation. Although this is applicable for several multivoltine butterfly species in the UK, there is even greater potential/relevance beyond the UK, for example in Europe where species may be multivoltine in warmer parts of their range. Models for multivoltine species may also have increasing relevance as climate warming may lead to increases in species' voltinism (Altermatt, 2010).

In future releases of the rGAI package, we intend to allow survival *ϕ* to vary with respect to spatial covariates, or within the season in terms of time or age (Matechou et al., 2014). There is also the potential to account for variation in detection probability, to reduce bias in estimates of relative abundance, using relevant covariates (Matechou et al., 2014). The package can also be extended for multi‐year fits and trend estimation; see for example Dennis et al. (2016). We also hope that researchers may contribute new developments to the package; for example accounting for skewness in patterns of seasonal variation/emergence would be of interest (Calabrese, 2012).

The GAI is also relevant for other taxa, for example birds on migration—see Matechou et al. (2013), beetles—see Dennis et al. (2021) who model the two‐year life cycle of fuliginators, *Iberodorcadion fuliginator*—and bees—see Matechou et al. (2018) who use a dynamic stopover model to analyze citizen science data on bumblebees, from the *BeeWalk* scheme. We anticipate that the rGAI package will enhance and enable further research by ecologists and applied statisticians, which can improve our understanding of changes in species' populations and phenology.

## AUTHOR CONTRIBUTIONS

**Emily B. Dennis:** Conceptualization (lead); data curation (lead); formal analysis (equal); software (equal); visualization (lead); writing – original draft (lead); writing – review and editing (equal). **Calliste Fagard‐Jenkin:** Software (equal); writing – review and editing (equal). **Byron J. T. Morgan:** Conceptualization (equal); writing – original draft (equal); writing – review and editing (equal).

## CONFLICT OF INTEREST

The authors declare no conflict of interest.

## Data Availability

The butterfly count data that feature in the examples are available in the rGAI package from https://github.com/calliste‐fagard‐jenkin/rGAI.
